# Vitamin E Enhances Immune Function and the Intestinal Histological Structure by Regulating the *Nodal*-Mediated Signaling Pathway: A Case Study on the Sea Cucumber *Apostichopus japonicus*

**DOI:** 10.3390/biology14081008

**Published:** 2025-08-06

**Authors:** Zitong Wang, Yan Wang, Xianyu Wang, Guangyao Zhao, Haiqing Zeng, Haoran Xiao, Lingshu Han, Jun Ding, Yaqing Chang, Rantao Zuo

**Affiliations:** Key Laboratory of Mariculture and Stock Enhancement in North China’s Sea (Ministry of Agriculture and Rural Affairs), Dalian Ocean University, Dalian 116023, China

**Keywords:** vitamin E, intestinal histological structure, *Nodal*, collagen, *Apostichopus japonicus*

## Abstract

The histological integrity of the intestine depends on the tight and orderly arrangement of epithelial cells within the intestinal villi. *Nodal*, a transforming growth factor-β (TGF-β) family member, has been reported to promote epithelial cell proliferation. Collagen not only establishes physical connections between adjacent cells but also serves as an anchoring platform for cell adhesion and regeneration processes. Therefore, a 21-day feeding trial was conducted using RNA interference to investigate the role of the *Nodal* gene in regulating intestinal collagen synthesis and histological structure integrity in juvenile *A. japonicus* fed diets containing graded levels of vitamin E (VE) (0, 200, and 400 mg/kg). The results showed that the addition of 200 mg/kg VE significantly improved the growth rate, immune enzyme activities and related gene expression, as well as intestinal villus morphology. Additionally, the addition of 200 mg/kg VE upregulated the expression of *Nodal*, which activated the expression of collagen synthesis-related genes and promoted collagen deposition in the intestines of *A. japonicus*. After *Nodal* gene knockdown, *A. japonicus* presented a decreased growth rate, damage to the intestinal histological structure, and impaired collagen synthesis, with the most notable findings observed in *A. japonicus* fed diets without VE addition. However, these detrimental effects were eliminated to some extent by the addition of 200 mg/kg VE. These findings indicate that VE improves immune function and intestinal histological structure in *A. japonicus* through a *Nodal*-dependent pathway.

## Simple Summary

Vitamin E (VE) improves intestinal histological structure and immune function in sea cucumber by promoting collagen synthesis through the *Nodal* gene. The addition of 200 mg/kg VE enhanced growth, immune function, and intestinal health. Knockdown of the *Nodal* gene led to impaired growth, intestinal damage, and reduced collagen synthesis, but these effects were partially reversed by VE. This study highlights the role of VE in improving intestinal health via the *Nodal* pathway.

## 1. Introduction

The demand for sea cucumber (*Apostichopus japonicus*), an important economic marine product, has increased in recent years. Overfishing has led to the depletion of wild *A. japonicus* resources, disrupting the ecological balance and threatening the sustainability of marine ecosystems [1]. *A. japonicus* farming has been found to effectively alleviate pressure on natural resources and protect marine ecosystems and biodiversity [2]. Seedling cultivation is considered crucial for sustainable aquaculture, as it ensures a stable supply of high-quality seedlings, reduces dependence on wild resources, and improves farming efficiency [3]. However, *A. japonicus* lacks adaptive immunity and relies entirely on innate defenses to cope with bacterial infections and environmental stressors under intensive culture conditions [4,5,6,7]. The frequent application of antibiotics in aquaculture has been associated with an increase in antibiotic resistance [8], exacerbated environmental pollution, and threats to food safety [9,10]. Therefore, developing effective nutritional strategies to enhance the immune response of *A. japonicus* is essential and offers a promising alternative to antibiotics.

Vitamin E (VE), a lipid-soluble antioxidant, has been shown to contribute significantly to the immune function of animals [11,12]. The beneficial immune modulation effects of VE include increasing the antioxidant capacity, inhibiting the expression of proinflammatory factors, and promoting the secretion of antimicrobial peptides [13,14]. In recent years, the intestine has been increasingly recognized as a key organ involved in immune regulation. The intestine was considered the first line of defense against pathogenic invasion [15]. The intestinal histological structure was typically assessed by morphological parameters such as villus height, villus width, and mucosal thickness, which were considered important indicators for evaluating the intestinal functional status of aquatic animals [15,16,17]. Well-developed villus structures and intact mucosal and muscular layers were believed to enhance absorptive efficiency and strengthen the physical barrier against pathogen adhesion and invasion [18,19]. Appropriate VE supplementation has been shown to improve intestinal histological structure in aquatic animals. The addition of VE at an appropriate level significantly increased the villus height and mucosal thickness of channel catfish (*Ictalurus punctatus*) [18] and the villus length, width, and surface area of Pompano (*Trachinotus ovatus*) [20]. However, the specific mechanisms by which VE regulates the intestinal histological structure remain insufficiently explored in aquatic animals.

The stability of the intestinal histological structure was dependent on the tight and orderly arrangement of epithelial cells, which was maintained by intercellular tight junctions (TJs) [21,22,23,24]. However, the stability of TJs was not solely influenced by intracellular regulatory mechanisms but also relied on the integrity and basal support of the intestinal villi [25,26]. The villi were primarily composed of collagen-rich connective tissue, which served as a structural matrix that not only provided physical support for epithelial cells but also functioned as an anchoring platform for cell adhesion and alignment [27]. In carnivorous teleosts, a densely organized collagen layer, known as the stratum compactum, was located beneath the mucosa, enhancing gut wall tension and preventing overdistension during feeding [28]. In addition, collagen was involved in establishing a stable extracellular matrix microenvironment that facilitated epithelial cell adhesion, migration, and renewal, which was essential for tissue regeneration [29]. One of the primary signaling pathways for collagen synthesis is the transforming growth factor-β (TGF-β) pathway [30]. Studies have demonstrated that collagen synthesis is induced by the activated TGF-β pathway in humans [31] and mice (*Mus musculus*) [32], and a similar effect has been observed in the body wall of *A. japonicus* [33]. However, the diversity of the TGF-β family has added complexity to the understanding of its regulatory role [34]. *Nodal*, a member of the TGF-β family, has been identified as a key regulator of inflammation and immune function [35,36,37]. In addition, *Nodal* has been reported to promote the proliferation of fibroblasts and bronchial epithelial cells [38,39]. Given that fibroblasts are the principal producers of collagen [40], it can be inferred that *Nodal* participates in the regulation of collagen synthesis.

Therefore, this study used RNA interference (RNAi) to explore the effects of VE on growth, immune function, intestinal histological structure, and relevant regulatory mechanisms in juvenile *A. japonicus*. The specific objectives of this study were to (1) analyze the effects of VE on the immune function and intestinal structure of juvenile *A. japonicus* and (2) elucidate the specific role of *Nodal* in regulating the intestinal histological structure from the perspective of collagen synthesis and immune regulation. These findings provide insights into nutritional strategies for enhancing the immune function of echinoderms, with potential applications in sustainable aquaculture practices.

## 2. Materials and Methods

### 2.1. Ethics Statement

*A. japonicus* was artificially bred in a hatchery located in Dalian, China. All experimental procedures in this study were performed in strict accordance with the relevant national guidelines of China and Dalian Ocean University.

### 2.2. Construction of the L4440-dsRNA-Nodal Expression System

To achieve knockdown of the *Nodal* gene in *A. japonicus*, the conserved sequences of the *Nodal* gene were first identified using the National Center for Biotechnology Information (NCBI) database. The primers used for amplifying the genes were designed using Primer 5.0 software (F: CCATATGCTGAGCTTCCCGT; R: GGATCCACTTACTCCAGCCG). Using cDNA derived from the intestine of *A. japonicus* as a template, polymerase chain reaction (PCR) amplification was performed, and the PCR products were subsequently sequenced (TianGen, Beijing, China). The sequencing results were compared to the *Nodal* gene sequence in the NCBI database to verify the accuracy of the amplified fragment.

The sequencing results were analyzed by using a small interfering RNA (siRNA) prediction website (http://sidirect2.rnai.jp/, accessed on 31 July 2025) to identify the siRNA target sites [41]. Finally, a 210 bp interference fragment containing four siRNA target sites was identified. The four siRNA target sequences were CACATCTCTTGGCATTGAAATTG; TGGGTATAGGATCCACTTACTCC; AGCCGATGTTTTGGAAATCAACC; and TACGAAGTTGCCTCTCTTTCTTC. The sequence of this interference fragment was synthesized chemically by Sangon Biotech Co., Ltd. (Shanghai, China) and inserted between the two T7 promoters in the L4440 vector (AngYubio, Shanghai, China).

Upon obtaining the desired plasmid, it was transferred into *E. coli HT115* competent cells (AngYubio, Shanghai, China). Single colonies were inoculated into 50 mL of LB liquid medium containing 100 mg/L ampicillin and 12.5 mg/L tetracycline (Solarbio, Beijing, China) and cultured until the optical density (OD) reached 0.5–0.6, followed by sequencing (TianGen, Beijing, China). Isopropyl β-D-1-thiogalactopyranoside (IPTG) (Solarbio, Beijing, China) was added to a final concentration of 0.5 mM, and expression was induced at 37 °C for 4 h to obtain the experimental culture [41]. The L4440 empty plasmid transferred into *HT115* served as the control group, which also underwent IPTG induction. IPTG-induced cultures were centrifuged at 7000× *g* for 10 min and resuspended in distilled water [42].

Double-stranded RNA (dsRNA) was extracted from the experimental group using the TRIzol (TianGen, Beijing, China) method, followed by digestion with deoxyribonuclease (DNase) and ribonuclease A (RNase A). The concentration of the dsRNA was measured to ensure its purity and integrity.

### 2.3. Diet Preparation

The feed formulation and manufacturing procedures have been described in one of our previous studies [33]. Three feeds were formulated by adding VE (sourced from Nanjing Dulai Biotechnology, with a purity of ≥96%) at concentrations of 0 mg/kg, 200 mg/kg, and 400 mg/kg, which were named the VE-deficient group (VE 0), VE-adequate group (VE 200), and VE-excessive group (VE 400), respectively. The feed ingredients and proximate compositions are provided in Table 1 to ensure clarity and consistency with our prior work [33].

### 2.4. Feeding Experiment

*A. japonicus* were artificially bred in a local hatchery in Dalian, China. Prior to the experiment, *A. japonicus* were acclimated to the experimental environments for two weeks and fed the VE 0 diet. A total of 180 healthy *A. japonicus* were evenly allocated into 12 rectangular 30 L tanks, with each tank housing 15 individuals, ensuring an initial total weight of approximately 195 ± 1 g.

Prior to feeding, a bacterial suspension containing 3.36 × 10^9^ CFUs was thoroughly mixed with each feed. The feed was supplemented with dsRNA at a final concentration of 6500 ng per kilogram of feed. To avoid dissolution, the mixture was kneaded into spherical feed pellets approximately 1.5 cm in diameter. In total, there were six feeding groups: VE 0 +ds, VE 0 +C, VE 200 +ds, VE 200 +C, VE 400 +ds, and VE 400 +C.

The juvenile *A. japonicus* used in this study were produced in May 2025 through artificial breeding. At the start of the experiment, the animals had an average initial body weight of approximately 12.7 ± 0.5 g. Rearing conditions during the experimental period were consistent with those maintained during the two-week acclimation phase. *A. japonicus* were fed twice daily (7:00 a.m. and 5:30 p.m.) at approximately 3% of their initial body weight. The remaining food and feces were removed before each feeding. On average, half of the water was exchanged every day. During the 21-day experimental period, the water temperature fluctuated between 18 °C and 19 °C, pH from 7.6 to 8.3, dissolved oxygen remained above 6 mg/L, and both ammonia nitrogen and nitrite levels were maintained below 0.05 mg/L [33].

### 2.5. Sample Collection

When the feeding experiment ended, the final body weights of all *A. japonicus* in each tank were individually measured after a 12 h fasting period. Then, 10 individuals were removed from each tank and dissected to collect coelomic fluid. The coelomic fluid was collected into 1.5 mL RNase-free centrifuge tubes (Axygen, Union City, CA, USA), centrifuged (4 °C, 10,000× *g*), and stored at −80 °C for enzyme activity analysis.

After that, the viscera and body wall were separated and weighed individually to calculate the body wall index (BWI). The intestine of each *A. japonicus* individual was carefully separated and straightened for imaging, and *A. japonicus* of the same size were used. After the remaining feces were gently removed, the length and weight of each sample were measured. Additionally, a 1 cm segment of the midgut of three experimental animals was fixed in 4% paraformaldehyde for 24 h (Sangon Biotech, Shanghai, China) for histological analysis. To avoid RNA and protein degradation, the remaining intestines were rapidly dissected on ice, washed with ice-cold PBS, collected into RNase-free centrifuge tubes, and immediately stored at −80 °C for the analysis of antioxidant enzyme activities and gene expression.

### 2.6. Physiological and Chemical Analysis

#### 2.6.1. Intestinal Histological Analysis

Intestinal samples were fixed in 4% paraformaldehyde, dehydrated, and cleared using a Leica ASP200S system (Leica Microsystems, Wetzlar, Germany), embedded in paraffin, and sectioned into 6 μm slices with a Leica RM2245 microtome. Sections were stained with hematoxylin and eosin (HE) following standard protocols: hematoxylin staining for 5 min, rinsing, eosin counterstaining for 2 min, followed by dehydration in graded ethanol, clearing in xylene, and mounting in neutral resin (Sangon Biotech, Shanghai, China). The stained sections were examined under a light microscope (Leica DM2500, Leica Microsystems, Wetzlar, Germany) to assess intestinal villus morphology and tissue integrity [43].

#### 2.6.2. Enzyme Activity Analysis

The intestinal tissues were homogenized and centrifuged, and the supernatant was collected for subsequent analysis [44]. The protein concentration was determined using the Bradford method. The activities of enzymes, including peroxidase (POD) (A084-1-1), glutathione peroxidase (GSH-Px) (A005-1-1), superoxide dismutase (SOD) (A001-1-1), malondialdehyde (MDA) (A003-1-2), acid phosphatase (ACP) (A060-1-1), alkaline phosphatase (ALP) (A059-2-2), lysozyme (LZM) (A050-1-1), and catalase (CAT) (A007-1-1), in the intestinal and coelomic fluids of *A. japonicus* were determined using commercial kits provided by Nanjing Jiancheng Institute (Nanjing, China).

#### 2.6.3. Collagen Content Analysis

The hydroxyproline (Hyp) content in the intestine of *A. japonicus* was measured using a kit (A030-2) (Nanjing Jiancheng Institute, Nanjing, China) following the manufacturer’s guidelines. The intestinal samples were prepared according to the kit instructions, and absorbance was measured at 550 nm using an Infinite^®^ Pro 200 microplate reader (Tecan, Männedorf, Switzerland) [33]. The collagen content was calculated from the standard curve supplied with the kit (AOAC, Rockville, MD, USA, 2002).

### 2.7. Real-Time Quantitative PCR

Total RNA was extracted from the intestine of *A. japonicus* using a TianGen RNA Easy Fast Kit (DP451), and its integrity was checked by agarose gel electrophoresis (Sangon Biotech, Shanghai, China). cDNA synthesis was subsequently carried out using FastKing gDNA Dispelling RT SuperMix (KR118) (TianGen, Beijing, China).

qPCR was conducted using the FastKing One Step RT—PCR Kit (KR123) (TianGen, Beijing, China), following the manufacturer’s recommended protocol [45]. The reaction system and cycling parameters were identical to those described in our previous study [33]. The sequences of primers used in this experiment are listed in Table 2. Relative expression levels were calculated using the 2^−ΔΔCT^ method. The results are expressed as the means ± standard errors (SEs) [46,47,48].

### 2.8. Formulas for Calculation

$$\mathrm{Survival}\;\mathrm{rate}\;(\mathrm{SR},\;\%)=N_{f}/N_{i}\times 100$$

$$\mathrm{Weight}\;\mathrm{growth}\;\mathrm{rate}\;(\mathrm{WGR},\;\%)=\left(W_{f}-W_{i}\right)/W_{i}\times 100$$

$$\mathrm{Specific}\;\mathrm{growth}\;\mathrm{rate}\;(\mathrm{SGR},\;\%)=\left\{\left[\ln\left(W_{f}\right)-\ln\left(W_{i}\right)\right]/T\right\}\times 100$$

$$\mathrm{Body}\;\mathrm{wall}\;\mathrm{index}\;(\mathrm{BWI}\%)=P_{W}/W\times 100$$

where $N_{f}$ and $N_{i}$ represent the final number and initial number, respectively; $W_{i}$, and $W_{f}$ represent the initial average weight and final weight of *A. japonicus* in each tank, respectively; $V_{W}$, $P_{W}$, and W represent the wet mass of the visceral organs, body wall index, and whole-body weight of *A. japonicus*, respectively; and T represents the feeding period.

### 2.9. Data Analysis

Statistical analyses were performed using SPSS 22.0. Data normality and homogeneity of variances were assessed using the Shapiro–Wilk and Levene’s tests, respectively. One-way analysis of variance (ANOVA) was used to assess the significance of differences among VE levels. When a significant difference (*p* < 0.05) was detected, Tukey’s test was performed for multiple comparisons to evaluate significant variations in mean values across different VE levels. Independent *t*-tests were conducted to compare the means under the same VE before and after suppression of *Nodal* expression at the same VE level. Villus length in the intestinal histological images was measured using Case Viewer software (version 2.4, 3DHISTECH Ltd., Budapest, Hungary). The results are presented as the means ± standard errors (SEs).

## 3. Results

### 3.1. Growth Performance

Before *Nodal* gene knockdown, the WGR and SGR of *A. japonicus* were significantly promoted by moderate addition of VE (200 mg/kg) (*p* < 0.05) but were inhibited by the highest addition level of VE (400 mg/kg) (Table 3). These growth indices were normalized to the individual body weight at the start of the feeding trial to account for size differences. After *Nodal* gene knockdown, the WGR and SGR of all the groups significantly decreased to different extents. Nonetheless, the highest WGR and SGR were observed in the moderate VE group, which were significantly greater than those in the VE 400 and VE 0 groups (*p* < 0.05). Notably, *A. japonicus* in the VE 0 group exhibited a negative growth rate (Table 3).

Before *Nodal* gene knockdown, the BWI of *A. japonicus* in the VE 200 group was slightly greater than that in the VE 400 and VE 0 groups (*p* > 0.05). After *Nodal* gene knockdown, the BWIs of the VE 0 and VE 400 groups were significantly lower (*p* < 0.05), with no significant differences found among the three groups (*p* > 0.05) (Table 3).

### 3.2. Intestinal Morphology

**Figure 1:**
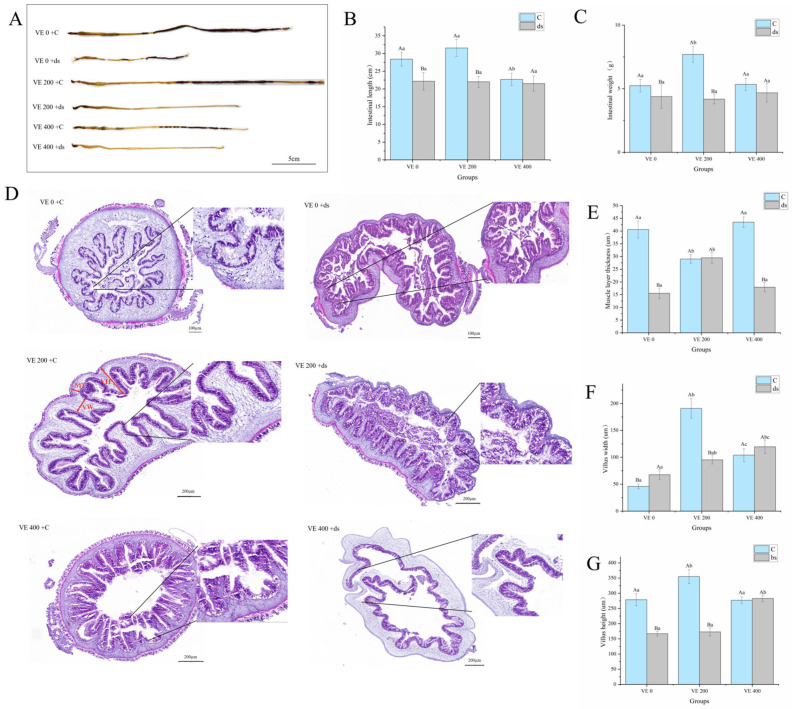
Effects of vitamin E (VE) on the intestinal morphology parameters in sea cucumbers (*Apostichopus japonicus*). (**A**) Intestinal morphology; (**B**) intestinal length; (**C**) intestinal weight; (**D**) intestinal tissue sections; (**E**) muscle layer thickness; (**F**) villus width; (**G**) villus height. Data are presented as mean ± SD (*n* = 3). Different uppercase letters (A/B) indicate statistically significant differences before and after *Nodal* knockdown at the same VE level (*p* < 0.05). Different lowercase letters (a/b/c) indicate significant differences among different VE levels (*p* < 0.05).

Histological analysis was performed using HE staining. Before *Nodal* gene knockdown, the intestinal length (IL) and weight (IW) of *A. japonicus* increased with moderate VE addition but declined at the highest VE level (Figure 1A–C). After *Nodal* gene knockdown, the ILs and IWs of *A. japonicus* in the VE 0 and VE 200 groups significantly decreased (*p* < 0.05). However, no significant differences were found among the three groups (*p* > 0.05) (Figure 1A–C).

Before *Nodal* gene knockdown, the intestinal morphology of *A. japonicus* exhibited closely arranged and continuous epithelial cells, in the group with moderate addition of VE. In contrast, the highest addition level of VE led to irregular, shortened, and partially broken villi, indicating a higher level of structural damage (Figure 1D). After *Nodal* gene knockdown, all groups exhibited varying degrees of villus rupture and epithelial shedding, with the most severe damage observed in the VE 0 group, which was characterized by extensive villus breakage. Nonetheless, the moderate VE group maintained the best villus integrity and continuous epithelial arrangement compared to the other groups (Figure 1D).

Before *Nodal* gene knockdown, the villus height and width of *A. japonicus* were significantly increased by moderate addition of VE (*p* < 0.05). The villus width was inhibited by the highest addition level of VE but remained significantly greater than that in the VE 0 group (*p* < 0.05) (Figure 1E–G). After *Nodal* gene knockdown, both the villus height and the villus width significantly decreased in the VE 200 group (*p* < 0.05). The muscle layer thickness in the VE 200 group did not significantly change (*p* > 0.05) (Figure 1E–G).

### 3.3. Immune Enzyme and Antioxidant Activities

#### 3.3.1. Coelomic Fluid Immune Enzyme and Antioxidant Activities

Before *Nodal* gene knockdown, the activities of antioxidant enzymes (SOD, GSH-Px, and CAT) in the coelomic fluid of *A. japonicus* were significantly increased by the moderate addition level of VE (*p* < 0.05) but inhibited by the highest addition level of VE. POD activity was highest in the VE 200 group, which was significantly greater than that in the VE 0 group (*p* < 0.05) (Table 4). After *Nodal* gene knockdown, the activities of SOD, GSH-Px, and CAT in all the groups significantly decreased to varying extents. Nonetheless, the highest levels of SOD, POD, and GSH-Px were observed in the VE 400 group, which were significantly greater than those in the VE 0 and VE 200 groups (*p* < 0.05) (Table 4).

Before *Nodal* gene knockdown, the MDA levels of *A. japonicus* significantly decreased with the moderate addition level of VE (*p* < 0.05) but significantly increased with the highest addition level of VE (Table 4). After *Nodal* gene knockdown, the MDA levels in all the groups significantly increased (*p* < 0.05). Nonetheless, the lowest MDA levels were observed in the moderate VE group, which were significantly greater than those in the VE 0 and VE 400 groups (*p* < 0.05) (Table 4).

Before *Nodal* gene knockdown, the activities of immune-related enzymes (LZM, ACP, and ALP) were significantly increased by moderate addition of VE (*p* < 0.05). However, ACP and ALP were inhibited by the highest addition level of VE (Table 4). After *Nodal* gene knockdown, the activities of LZM, ALP, and ACP in all the groups significantly decreased (*p* < 0.05). Nonetheless, the highest levels of LZM and ACP were observed in the moderate VE group, which were significantly greater than those in the VE 400 and VE 0 groups (*p* < 0.05). ALP activity was significantly reduced in the VE 0 and VE 200 groups, with the highest level again observed in the moderate VE group (Table 4)

#### 3.3.2. Intestinal Immune Enzyme and Antioxidant Activities

Before *Nodal* gene knockdown, the activities of antioxidant enzymes (SOD, GSH-Px, and CAT) in the intestine of *A. japonicus* significantly increased with the moderate addition level of VE (*p* < 0.05) but were inhibited by the highest addition level of VE (Table 5). After *Nodal* gene knockdown, the activities of SOD, GSH-Px, and CAT in all the groups significantly decreased (*p* < 0.05). No significant changes in POD activity were observed in the VE 400 group (*p* > 0.05). The highest levels of SOD, POD, and GSH-Px were still observed in the VE 400 group, and the highest level of CAT was still observed in the VE 200 group (*p* < 0.05) (Table 5).

Before *Nodal* gene knockdown, the MDA levels of *A. japonicus* significantly decreased with the moderate addition level of VE (*p* < 0.05) but significantly increased with the highest addition level of VE (*p* < 0.05) (Table 5). After *Nodal* gene knockdown, the MDA levels in all the groups significantly increased (*p* < 0.05). Nonetheless, the MDA levels remained lowest in the moderate VE group, and significantly higher in the VE 400 and VE 0 groups (*p* < 0.05) (Table 5).

Before *Nodal* gene knockdown, the activities of immune-related enzymes (LZM, ACP, and ALP) were increased by the moderate addition level of VE but were inhibited by the highest addition level of VE (Table 5). After *Nodal* gene knockdown, the LZM, ACP, and ALP activities in all the groups significantly decreased to varying extents (*p* < 0.05). Nonetheless, the highest levels of LZM and ACP were observed in the VE 400 group, whereas the highest level of ALP was observed in the VE 200 group (*p* < 0.05) (Table 5).

### 3.4. Intestinal Collagen Synthesis

**Figure 2:**
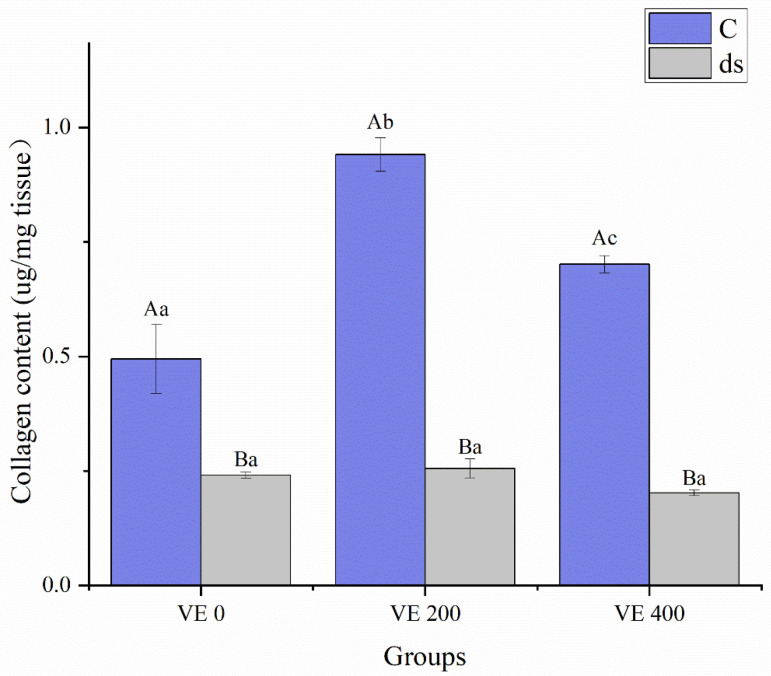
Effects of vitamin E (VE) on the collagen content in the intestine of sea cucumbers (*Apostichopus japonicus*). Data are presented as mean ± SD (*n* = 3). Different uppercase letters (A/B) indicate statistically significant differences before and after *Nodal* knockdown at the same VE level (*p* < 0.05). Different lowercase letters (a/b/c) indicate significant differences among different VE levels (*p* < 0.05).

Before *Nodal* gene knockdown, the collagen content of *A. japonicus* was significantly increased by the moderate addition level of VE (*p* < 0.05) but inhibited by the highest addition level of VE, although it remained significantly greater than that in the VE 0 group (*p* < 0.05) (Figure 2). After *Nodal* gene knockdown, collagen content declined significantly across all groups (*p* < 0.05), with no significant differences among them (*p* > 0.05) (Figure 2).

**Figure 3:**
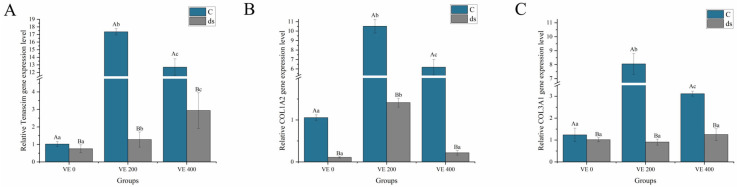
Effects of vitamin E (VE) on the expression of collagen-related genes in the intestine of sea cucumbers (*Apostichopus japonicus*). (**A**) *Tenascin*; (**B**) *COL1A2*; (**C**) *COL3A1*. Data are presented as mean ± SD (*n* = 3). Different uppercase letters (A/B) indicate statistically significant differences before and after *Nodal* knockdown at the same VE level (*p* < 0.05). Different lowercase letters (a/b/c) indicate significant differences among different VE levels (*p* < 0.05).

Before *Nodal* gene knockdown, the levels of the collagen-related genes tenascin-c (tenascin), collagen type I alpha 2 chain (*COL1A2*), and collagen type III alpha 1 chain (*COL3A1*) in the intestine of *A. japonicus* were significantly increased by the moderate addition level of VE (*p* < 0.05) but inhibited by the highest addition level of VE (*p* < 0.05) (Figure 3). After *Nodal* gene knockdown, the tenascin, *COL1A2*, and *COL3A1* levels in all the groups significantly decreased to varying extents. Nonetheless, the highest levels of *COL1A2* were observed in the moderate VE group. No significant differences were observed in the levels of tenascin among the three groups (*p* > 0.05) (Figure 3).

### 3.5. Gene Expression

#### 3.5.1. *Nodal* and SMAD Gene Expression

**Figure 4:**
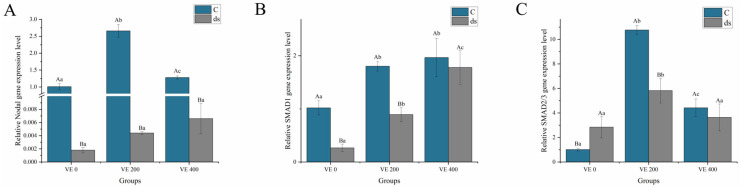
Effects of vitamin E (VE) on the expression of *Nodal* and SMAD in the intestine of sea cucumbers (*Apostichopus japonicus*). (**A**) *Nodal*; (**B**) *SMAD1*; (**C**) *SMAD2/3*. Data are presented as mean ± SD (*n* = 3). Different uppercase letters (A/B) indicate statistically significant differences before and after *Nodal* knockdown at the same VE level (*p* < 0.05). Different lowercase letters (a/b/c) indicate significant differences among different VE levels (*p* < 0.05).

Before *Nodal* gene knockdown, the levels of *Nodal*, sp-*SMAD2/3* (*SMAD2/3*), and Sma- and Mad-related protein (*SMAD1*) in the intestine of *A. japonicus* were significantly increased by moderate addition of VE (*p* < 0.05). However, *Nodal* and *SMAD2/3* were inhibited by the highest addition level of VE (*p* < 0.05) (Figure 4). After *Nodal* gene knockdown, the *Nodal* and *SMAD1* levels in all the groups significantly decreased to varying extents. The silencing efficiencies of the *Nodal* gene were calculated based on the relative expression levels between the control and dsRNA groups, and reached 99.82%, 99.83%, and 99.48% in the VE 0, VE 200, and VE 400 groups, respectively (Figure 4A). *SMAD2/3* levels were reduced in both the VE 200 and VE 400 groups. The highest level of *Nodal* was observed in the VE 400 group, whereas the highest levels of *SMAD2/3* and *SMAD1* were observed in the VE 200 group. Notably, after *Nodal* gene knockdown, *SMAD2/3* levels were significantly increased in the VE 200 group (*p* < 0.05) (Figure 4).

#### 3.5.2. Immune-Related Gene Expression

**Figure 5:**
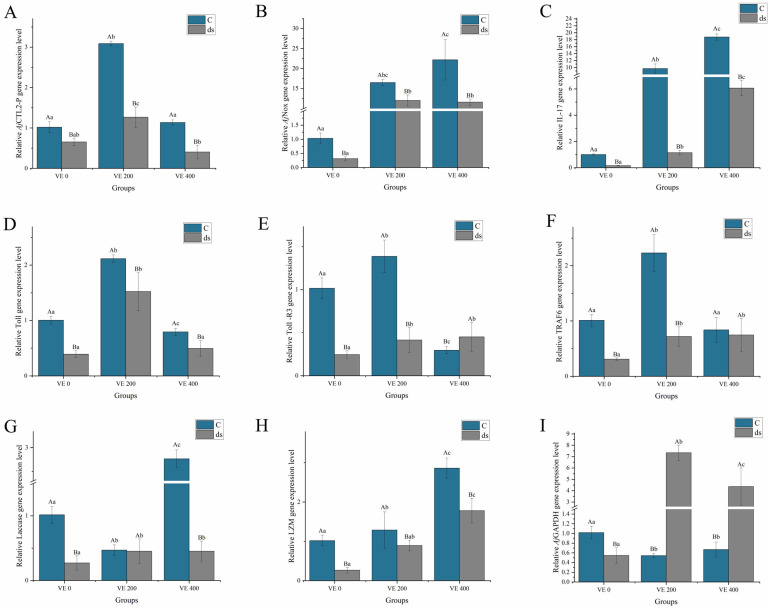
Effects of vitamin E (VE) on the expression of immune-related genes in the intestine of sea cucumbers (*Apostichopus japonicus*). (**A**) *AjCTL2-p*; (**B**) *AjNox*; (**C**) *IL-17*; (**D**) *Toll*; (**E**) *Toll-R3*; (**F**) *TRAF6*; (**G**) *Laccase*; (**H**) *LZM*; (**I**) *AjGAPDH*. Data are presented as mean ± SD (*n* = 3). Different uppercase letters (A/B) indicate statistically significant differences before and after *Nodal* knockdown at the same VE level (*p* < 0.05). Different lowercase letters (a/b/c) indicate significant differences among different VE levels (*p* < 0.05).

Before *Nodal* gene knockdown, the expression of the immune-related genes *A. japonicus* C-type lectin 2 precursor (*AjCTL2-p*), *Toll*, Toll-like receptor 3 (*Toll-R3*), and tumor necrosis factor receptor-associated factor 6 (*TRAF6*) in the intestine of *A. japonicus* was significantly increased by the moderate addition of VE (*p* < 0.05). The *A. japonicus* NADPH oxidase (*AjNox*), *IL-17*, *Laccase*-type phenoloxidase (*Laccase*), and lysozyme (*LZM*) levels were significantly increased by the highest addition level of VE (*p* < 0.05) (Figure 5).

After *Nodal* gene knockdown, the expression of *AjCTL2-p*, *AjNox*, *IL-17*, *Toll*, *TRAF6*, *Laccase*, and *LZM* in all the groups significantly decreased to varying extents. The highest levels of *AjCTL2-p* and *Toll* were observed in the VE 200 group, which were significantly greater than those in the VE 400 and VE 0 groups (*p* < 0.05). The highest levels of *LZM* were observed in the VE 400 group, which were significantly greater than those in the VE 0 and VE 200 groups (*p* < 0.05). No significant differences were found in the expression of the other genes among the three groups (*p* > 0.05). Notably, the expression of *A. japonicus* glyceraldehyde-3-phosphate dehydrogenase (*AjGAPDH*) significantly increased after knockdown, reaching its highest level in the VE 200 group (*p* < 0.05) (Figure 5).

## 4. Discussion

VE is widely recognized for its beneficial role in enhancing immune function and promoting the growth of aquatic animals [49]. The requirements for VE have been quantified in a variety of aquatic animals [50]. The addition of 80 mg/kg VE significantly accelerated the growth rate of discus fish (*Symphysodon haraldi*) [51], whereas 160 mg/kg VE increased both the growth rate and immune function of male oriental river prawns (*Macrobrachium nipponense*) [52]. In this study, the WGR and SGR of *A. japonicus* were significantly increased by the addition of 200 mg/kg VE. Moreover, the activities of immune enzymes (LZM, ACP, and ALP) as well as the expression of immune genes (*AjCTL2-p*, *AjNox*, *IL-17*, *Toll*, *Toll-R3*, *TRAF6*) were markedly increased by the addition of 200 mg/kg VE. This finding was consistent with the findings of several previous studies showing that the addition of VE at a concentration of 100–200 mg/kg significantly promoted the growth and enhanced the nonspecific immune responses of *A. japonicus* [33,53].

*A. japonicus*, an invertebrate that relies on innate immunity, depends on the intestine as its primary immune organ [54]. The intestine has been reported to prevent the invasion of pathogens and harmful substances [55]. From a histomorphological perspective, parameters such as intestinal length, villus height, and villus width are critical indicators of intestinal histological structure and functional capacity. Numerous studies have demonstrated a positive correlation between these histological parameters and both intestinal health status and nutrient absorption efficiency in aquatic species [56,57]. Intestinal villus atrophy is commonly regarded as an indicator of intestinal diseases in aquatic animals [58,59]. Nutrients, at appropriate levels, have beneficial effects on the intestinal health of animals [60]. Among them, VE is attracting increasing attention because of its multiple physiological effects on the enhancement of the intestinal histological structure. Specifically, dietary addition of 50–100 mg/kg VE significantly increased the intestinal length of channel catfish (*Ictalurus punctatus*) [18] and improved the villus height and width in pompano (*Trachinotus ovatus*) [20] and Nile tilapia (*Oreochromis niloticus*) [61]. These improvements not only suggest structural optimization but also imply enhanced absorptive surface area and improved nutrient uptake capacity. In this study, intestinal length and weight, along with villus height and width, were significantly increased by the addition of 200 mg/kg VE. These results not only corroborated observations reported in other aquatic species but also emphasized that dietary VE supplementation was capable of optimizing intestinal morphological development in *A. japonicus*, particularly with respect to intestinal length, villus height, and villus width.

Intestinal histological integrity, a critical parameter of intestinal health [55,62], includes both cellular integrity and the integrity of intercellular junctions [63]. Cellular integrity is protected from lipid peroxidation-induced oxidative stress by a series of antioxidant enzymes [64]. VE, at appropriate levels, was found to protect cellular integrity by preventing lipid peroxidation in cell membranes [11]. In this study, the addition of 200 mg/kg VE resulted in the most intact villus morphology in the intestine of *A. japonicus*. Consistently, the antioxidant capacity, reflected by the activities of antioxidant enzymes (SOD, GSH-Px, and CAT), was increased by the addition of 200 mg/kg VE. A positive relationship between the antioxidant capacity and structural integrity of the intestinal epithelium was also observed in swimming crabs (*Portunus trituberculatus*) [65] and Nile tilapia (*Oreochromis niloticus*) [61]. However, although VE was widely recognized as an effective antioxidant, its excessive intake was also found to produce adverse effects [33]. When administered at high levels, VE was reported to promote the generation of reactive oxygen species (ROS) through redox cycling mechanisms, which in turn induced oxidative stress, disrupted cellular membranes and organelles, and ultimately led to structural damage of the intestinal tissue [66]. In this study, evident intestinal damage and a significant reduction in antioxidant capacity were observed in the group treated with 400 mg/kg VE. Similarly, in grass carp (*Ctenopharyngodon idellus*) and Nile tilapia (*Oreochromis niloticus*), excessive dietary supplementation with VE was also reported to impair the endogenous antioxidant enzyme system and cause muscle atrophy [19,61,67]. In summary, appropriate supplementation with VE was found to effectively maintain the intestinal cellular integrity of *A. japonicus* by enhancing antioxidant enzyme activity and reducing oxidative stress, whereas excessive supplementation was shown to cause tissue damage, further confirming the dose-dependent effects of VE.

The integrity of intercellular junctions plays a critical role in maintaining intestinal histological integrity, as reflected by the tight alignment of epithelial cells [63,68]. The integrity of intercellular junctions is critically dependent on cell adhesion and cell renewal [69,70,71]. Cell adhesion and cell renewal are maintained by collagen [72,73]. Collagen not only establishes physical connections between adjacent cells but also serves as an anchoring platform for cell adhesion and regeneration processes [74,75]. Type I collagen was shown to facilitate cell adhesion via integrin β1 and neural cadherin [76] while also activating intracellular signaling pathways through integrin α1β1, ultimately promoting cytoskeletal organization and cell renewal [77]. Similarly, intercellular junction integrity was found to be positively correlated with the collagen content of the muscle of the spotted mackerel (*Scomber australasicus*) [78] and Japanese sea bass (*Lateolabrax japonicus*) [79]. In this study, the intestinal collagen content significantly increased following the addition of 200 mg/kg VE. This finding indicates that the appropriate addition of VE was helpful for maintaining intercellular junction integrity. Histological analysis further demonstrated that the highest degree of structural preservation was exhibited by intestinal villi in the group administered 200 mg/kg VE. In an earlier investigation, it was reported that collagen production in the body wall of *A. japonicus* was markedly enhanced by VE supplementation. [33]. As mentioned above, antioxidant capacity plays an important role in maintaining intestinal intercellular junction integrity [80]. Collagen was confirmed to possess antioxidant and anti-inflammatory properties in previous studies [81,82,83]. Thus, collagen can promote intestinal intercellular junction integrity by alleviating oxidative stress [80] and inflammation [82,84].

**Figure 6:**
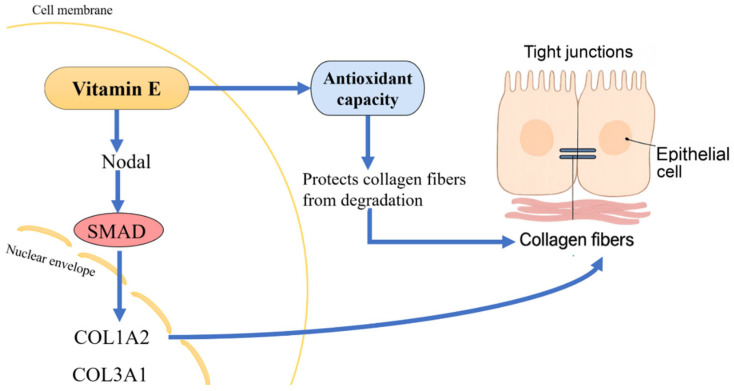
Schematic diagram of the proposed mechanism by which Vitamin E promotes collagen synthesis and enhances intestinal histological structure via the *Nodal*–SMAD pathway.

Collagen synthesis is regulated primarily by the TGF-β signaling pathway [30]. The TGF-β superfamily comprises numerous members with complex functions [85]. Within the TGF-β superfamily, *Nodal* has recently garnered attention for its critical role in embryonic patterning and epithelial renewal [38,86]. During the process of cell regeneration, collagen has been proven to be essential [82]. Moreover, *Nodal* was demonstrated to promote the proliferation of fibroblasts, which were identified as the main source of collagen production [38,87]. The *Nodal* signaling pathway is transmitted via the phosphorylation of SMAD proteins [86]. Specifically, *Nodal* binding to its receptor activates *SMAD2/3* proteins, which then form complexes with SMAD4 and translocate into the nucleus, where they promote the transcription of collagen-related genes (such as *COL1A2* and *COL3A1*) and other extracellular matrix components [86]. This activation of the SMAD pathway has been shown to increase collagen synthesis in aquatic species such as yellow croaker (Nibea coibor) [88] and grass carp (Ctenopharyngodon idella) [89]. Consistent with these findings, our study found that supplementation with 200 mg/kg of VE was associated with increased *Nodal* gene expression. Furthermore, the expression levels of *Nodal* and its downstream targets (*SMAD1* and *SMAD2/3*) were significantly upregulated in the 200 mg/kg VE group. Histological observations further confirmed that this group exhibited the most intact villus morphology and intercellular junction integrity. These results suggest that VE may promote collagen synthesis in A. japonicus through activation of the *Nodal*/SMAD pathway, integrating its antioxidant properties with transcriptional regulation of extracellular matrix components. Taken together, these findings confirm that VE promotes collagen synthesis in A. japonicus through activation of the *Nodal*/SMAD signaling cascade (Figure 6).

In this study, RNAi experiments were performed to further verify whether *Nodal* is involved in the VE-mediated regulation of growth and intestinal histological structure. *A. japonicus* is highly sensitive to external stimuli and prone to stress-induced evisceration [90], making it unsuitable for invasive procedures such as injection-based RNAi. Although efficient, injection methods carry a risk of visceral injury and increased mortality in this species [91,92]. Bacteria-mediated dsRNA delivery, a noninvasive gene silencing approach, has been tested in several teleost fish species [41]. Thus, a feeding-based bacteria-mediated dsRNA delivery system is considered a safer and more efficient RNAi method for *A. japonicus* [91,93]. In this study, *Nodal* expression was significantly suppressed, with no mortality observed across all groups during the entire experimental period, further confirming the effectiveness of the gene silencing method. Furthermore, *Nodal* knockdown led to negative growth, reduced intestinal collagen content, downregulation of key collagen genes (*COL1A2* and *COL3A1*), downregulation of immune-related genes, and histological evidence of intestinal injury. These results highlight the importance of *Nodal* in regulating growth, collagen synthesis, immune function, and intestinal histological structure. The overexpression of *AjGAPDH* following *Nodal* silencing was observed, but its biological relevance remains unclear. The upregulation of *AjGAPDH* was attributed to a nonspecific stress response induced by RNAi, or to a compensatory mechanism triggered by metabolic or redox imbalance after *Nodal* silencing [94]. *Nodal* can regulate the proliferation and differentiation of epithelial cells [38]. This observation was further supported by our findings, where severe intestinal injury was observed following *Nodal* knockdown. The damage was presumably linked to decreased collagen levels, which may have compromised the structural support necessary for epithelial cohesion, regenerative capacity, and barrier integrity. Additionally, previous studies have shown that collagen plays a crucial role in maintaining the normal permeability of the intestine by promoting the formation of TJs, with intestinal permeability being an important indicator of intestinal barrier function [95,96]. Although functional indicators of intestinal barrier integrity were not directly assessed in this study, the intestinal morphological results suggest that *Nodal* gene knockdown may have indirectly disrupted intestinal barrier function by affecting collagen synthesis. Future studies should focus on evaluating intestinal permeability and tight junction proteins as functional indicators to further validate this hypothesis.

## 5. Conclusions

In conclusion, the dietary addition of 200 mg/kg VE significantly enhanced growth performance, immune function, and intestinal histological structure in juvenile *A. japonicus*. After *Nodal* gene knockdown, *A. japonicus* exhibited a decreased growth rate, intestinal structure damage, and impaired collagen synthesis, with the most notable findings observed in *A. japonicus* fed diets without VE addition. However, these detrimental effects were partially alleviated by the addition of 200 mg/kg VE. Thus, the beneficial effects of VE on intestinal function could be attributed to enhanced intestinal antioxidant capacity and collagen synthesis through a *Nodal*-dependent pathway. This study highlights the importance of optimizing nutritional strategies to improve intestinal function in *A. japonicus*. Further research is needed to explore the effects of other nutritional immune stimulants and their interactions on the intestinal function of *A. japonicus.*

## Figures and Tables

**Table 1:** Ingredients and proximate analysis of the experimental diets (g/kg) [33].

Ingredients	Vitamin E Levels (mg/kg)		
	0 mg/kg	200 mg/kg	400 mg/kg
Fish meal	40	40	40
Fermented soybean meal	50	50	50
*Sargassum thunbergii* meal	290	290	290
Fish oil	10	10	10
Vitamin premix	5	5	5
DL-α-Tocopherol acetate (97% purity)	-	0.2	0.4
Mineral premix	5	5	5
Sea mud	600	599.8	599.6
Proximate composition			
Crude protein (%)	8.48 ± 0.29	8.39 ± 0.12	8.41 ± 0.32
Crude lipid (%)	0.53 ± 0.09	0.55 ± 0.01	0.58 ± 0.01

**Table 2:** Real-time PCR primers used in the present study.

Genes	Primer Sequences (5′-3′)	Gene IDs/References
*Cytb-R* ^1^	F:5′-TGAGCCGCAACAGTAATC-3′	KP170618.1
	R:5′-AAGGGAAAAGGAAGTGAAAG-3′	
*Tenascin* ^2^	F:5′-CCCTGATGGTGCTCT-3′	c64081.graph_c1
	R:5′-GGGACGAATCTTCATTTCTGTA-3′	
*COL1A2* ^3^	F:5′-CGGACTTTTACTTTGGCGTTAT-3′	c54738.graph_c0
	R:5′-TTTCTGGCGGTCTGCCTAT-3′	
*COL3A1* ^4^	F:5′-TCTCTTTGGTCACGATTGGC-3′	BSL78_04953
	R:5′-CACCACGGGCATCTGTTAG-3′	
*SMAD2/3* ^5^	F:5′-CAGAACCACCACGAACTCAA-3′	BSL78_11878
	R:5′-TGACTCACAGATACCACGGT-3′	
*SMAD1* ^6^	F:5′-ACACATTTACTTGGCTCCCC-3′	BSL78_15508
	R:5′-GTCCAGTTGTGAAGAGGCTT-3′	
*Nodal* ^7^	F:5′-GTTGTCTACACGAAGGGGTC-3′	BSL78_20284
	R:5′-CTGCATTTCAACCACACGAC-3′	
*AjCTL2-p*	F:5′-ACCGCTCCTCACCCTTTAACAC-3′	[47]
	R:5′-CCACTAGCACTAAGCAGCATATCAG-3′	
*AjNox*	F:5′-TAGCCCAAGAAAATCAAGGAGAA-3′	[48]
	R:5′-CAGCATTTGTGAAGGATGTGTGA-3′	
*IL-17*	F:5′-ACCGCTCCTCACCCTTTAACAC-3′	
	R:5′-ACACAAACCTGCCCTACATCA-3′	
*AjGAPDH*	F:5′-GGCATTCCGTGTACCTGTCCC-3′	
	R:5′-TACTGCTGGCTGCTTTTTTGA-3′	
*Laccase* ^8^	F:5′-TGTAGGGCATAATCAACCGG-3′	BSL78_13504
	R:5′-CAAACCTCTCCCCTGCATTG-3′	
*LZM* ^9^	F:5′-TCCTCTTCCCTAGCTCTACA-3′	BSL78_07135
	R:5′-AGTGAATGGCGATGTTGGTC-3′	
*Toll* ^10^	F:5′-CCGGGTTACATGTCACTGTT-3′	BSL78_01296
	R:5′-ACAACCCATTCAACTGCACT-3′	
*Toll-R3* ^11^	F:5′-TTATCGAGAACATCACCGGC-3′	BSL78_17684
	R:5′-GCCTCCTTCAACTTTTCCCA-3′	
*TRAF6* ^12^	F:5′-CTTCCGTTTCAAGCAGTCCT-3′	BSL78_15285
	R:5′-ATAACATTGTCGAGTGGCCC-3′	

^1^ *Cytb-R*, cytochrome b reductase. ^2^ *Tenascin*, tenascin-c. ^3^ *COL1A2*, collagen type I alpha 2 chain. ^4^ *COL3A1*, collagen type III alpha 1 chain. ^5^ *SMAD2/3*, sp-*SMAD2/3*. ^6^ *SMAD1*, Sma- and Mad-related protein 1. ^7^ *Nodal*, transforming growth factor beta family member *Nodal*. ^8^ *Laccase*, *Laccase*-type phenoloxidase. ^9^ *LZM*, lysozyme. ^10^ *ToII*, *Toll*. ^11^ *Toll-R3*, *Toll*-like receptor 3. ^12^ *TRAF6*, tumor necrosis factor receptor-associated factor 6.

**Table 3:** Effects of vitamin E (VE) on the growth performance of sea cucumber (*Apostichopus japonicus*).

	VE 0		VE 200		VE 400	
	C ^1^	Ds ^2^	C	ds	C	ds
Survival rate (%)	100	100	100	100	100	100
Weight growth rate (%)	55.22 ± 0.04 ^Aa^	−12.28 ± 0.02 ^Ba^	88.93 ± 0.02 ^Ab^	32.26 ± 0.05 ^Bb^	52.47 ± 0.03 ^Ac^	15.55 ± 0.01 ^Bc^
Specific growth rate (%/d)	2.09 ± 0.01 ^Aa^	−0.63 ± 0.01 ^Ba^	3.03 ± 0.01 ^Ab^	1.32 ± 0.02 ^Bb^	2.01 ± 0.01 ^Aa^	0.69 ± 0.01 ^Bc^
Body wall index (%)	63.11 ± 1.22 ^Aa^	52.71 ± 3.81 ^Ba^	64.36 ± 1.56 ^Aa^	61.29 ± 1.83 ^Ba^	59.68 ± 1.12 ^Aa^	59.19 ± 2.33 ^Aa^

^1^ C: control group; ^2^ ds: *Nodal* knockdown group. Data are presented as mean ± SD (*n* = 3). Different uppercase letters (A/B) indicate statistically significant differences before and after *Nodal* knockdown at the same VE level (*p* < 0.05). Different lowercase letters (a/b/c) indicate significant differences among different VE levels (*p* < 0.05).

**Table 4:** Effects of vitamin E (VE) on the immune enzyme and antioxidant activities in the coelomic fluid of sea cucumber (*Apostichopus japonicus*).

	VE 0		VE 200		VE 400	
	C ^1^	Ds ^2^	C	ds	C	ds
SOD (U/mL)	202.37 ± 5.94 ^Aa^	195.75 ± 1.09 ^Ba^	266.10 ± 0.70 ^Ab^	219.78 ± 3.94 ^Bb^	253.78 ± 2.74 ^Ac^	231.04 ± 2.63 ^Bc^
POD (U/mL)	0.65 ± 0.01 ^Aa^	0.44 ± 0.19 ^Ba^	2.19 ± 0.22 ^Ab^	1.39 ± 0.08 ^Bb^	1.92 ± 0.18 ^Ab^	1.96 ± 0.10 ^Ac^
GSH-P_X_ (U/mL)	6.73 ± 0.24 ^Aa^	6.36 ± 0.28 ^Ba^	15.70 ± 0.16 ^Ab^	8.88 ± 0.57 ^Bb^	11.52 ± 1.09 ^Ac^	10.91 ± 0.01 ^Bc^
CAT (U/mL)	0.38 ± 0.07 ^Aa^	0.32 ± 0.09 ^Aa^	0.72 ± 0.05 ^Ab^	0.32 ± 0.08 ^Ba^	0.17 ± 0.02 ^Bc^	0.20 ± 0.06 ^Aa^
MDA (nmol/mL)	0.77 ± 0.03 ^Aa^	0.59 ± 0.05 ^Ba^	0.24 ± 0.02 ^Bb^	0.33 ± 0.01 ^Ab^	0.97 ± 0.02 ^Bc^	1.19 ± 0.02 ^Ac^
LZM (U/mL)	56.15 ± 2.70 ^Aab^	39.74 ± 1.36 ^Ba^	102.56 ± 3.98 ^Ac^	51.79 ± 0.68 ^Bb^	58.72 ± 0.26 ^Aa^	33.59 ± 2.45 ^Bc^
ACP (King’s units/100 mL)	0.55 ± 0.02 ^Aab^	0.50 ± 0.03 ^Ba^	1.08 ± 0.04 ^Ac^	0.59 ± 0.01 ^Bab^	0.96 ± 0.08 ^Ab^	0.68 ± 0.06 ^Bc^
ALP (King’s units/100 mL)	0.96 ± 0.03 ^Aa^	0.44 ± 0.08 ^Ba^	2.71 ± 0.21 ^Ab^	1.40 ± 0.12 ^Bb^	1.21 ± 0.14 ^Ac^	1.06 ± 0.04 ^Bc^

^1^ C: control group; ^2^ ds: *Nodal* knockdown group. Data are presented as mean ± SD (*n* = 3). Different uppercase letters (A/B) indicate statistically significant differences before and after *Nodal* knockdown at the same VE level (*p* < 0.05). Different lowercase letters (a/b/c) indicate significant differences among different VE levels (*p* < 0.05).

**Table 5:** Effects of vitamin E (VE) on the immune enzyme and antioxidant activities in the intestine of sea cucumber (*Apostichopus japonicus*).

	VE 0		VE 200		VE 400	
	C ^1^	Ds ^2^	C	ds	C	ds
SOD (U/mgprot)	348.90 ± 0.44 ^Aa^	285.33 ± 3.84 ^Ba^	451.33 ± 4.69 ^Ab^	364.36 ± 2.07 ^Bb^	340.73 ± 3.18 ^Aa^	277.80 ± 1.48 ^Ba^
POD (U/mgprot)	5.52 ± 0.54 ^Aa^	4.70 ± 0.21 ^Ba^	10.34 ± 0.54 ^Ab^	6.52 ± 0.33 ^Bb^	4.44 ± 0.66 ^Aa^	4.94 ± 0.67 ^Aab^
GSH-PX (U/mgprot)	15.23 ± 0.69 ^Aab^	12.20 ± 0.28 ^Ba^	26.30 ± 1.69 ^Ac^	16.04 ± 1.30 ^Bb^	16.18 ± 1.67 ^Ab^	9.68 ± 0.85 ^Bc^
CAT (U/mgprot)	66.69 ± 4.52 ^Aab^	46.36 ± 8.86 ^Ba^	113.99 ± 1.20 ^Ac^	34.08 ± 5.39 ^Bc^	72.35 ± 4.28 ^Ab^	40.84 ± 1.43 ^Bab^
MDA (nmol/mgprot)	1.06 ± 0.15 ^Ba^	2.84 ± 0.30 ^Aa^	0.46 ± 0.22 ^Bb^	1.75 ± 0.34 ^Ab^	2.00 ± 0.06 ^Bc^	2.30 ± 0.07 ^Aab^
LZM (U/mgprot)	59.00 ± 2.10 ^Aa^	33.77 ± 3.83 ^Ba^	98.95 ± 0.95 ^Ab^	39.59 ± 1.38 ^Bb^	33.23 ± 1.65 ^Ac^	29.43 ± 1.03 ^Bc^
ACP (King’s units/gprot)	113.49 ± 2.16 ^Aa^	107.30 ± 1.03 ^Ba^	219.69 ± 15.20 ^Ac^	150.49 ± 14.25 ^Bb^	130.52 ± 2.90 ^Aab^	114.13 ± 9.44 ^Ba^
ALP (King’s units/gprot)	1078.08 ± 17.06 ^Aa^	1028.65 ± 15.64 ^Aa^	1372.85 ± 85.32 ^Ab^	499.07 ± 87.84 ^Bb^	1307.32 ± 80.93 ^Aab^	1133.30 ± 82.36 ^Bc^

^1^ C: control group; ^2^ ds: *Nodal* knockdown group. Data are presented as mean ± SD (*n* = 3). Different uppercase letters (A/B) indicate statistically significant differences before and after *Nodal* knockdown at the same VE level (*p* < 0.05). Different lowercase letters (a/b/c) indicate significant differences among different VE levels (*p* < 0.05).

## Data Availability

The original contributions presented in this study are included in the article. Further inquiries can be directed to the corresponding author.
